# Impact of availability of guidelines and active surveillance in reducing the incidence of ventilator-associated pneumonia in Europe and worldwide

**DOI:** 10.1186/1471-2334-14-199

**Published:** 2014-04-12

**Authors:** Klaus Kaier, Marie-Laurence Lambert, Uwe K Frank, Werner Vach, Martin Wolkewitz, Evelina Tacconelli, Jordi Rello, Ursula Theuretzbacher, Maria Martin

**Affiliations:** 1 Institute of Medical Biometry and Medical Informatics, University Medical Center, Freiburg, Germany; 2 Department of Environmental Health Sciences, University Medical Center, Freiburg, Germany; 3 Scientific Institute of Public Health, Brussels, Belgium; 4 Division of Infection Control and Hospital Epidemiology, Department of Infectious Diseases, Heidelberg University Hospital, Heidelberg, Germany; 5 Infectious Diseases, Department of Internal Medicine I, University Hospital, Tübingen, Germany; 6 Critical Care Department. Hospital Vall d’Hebron. Barcelona, Spain and CIBER de Epidemiología y Salud Pública (CIBERESP), Universidad Autónoma de Barcelona, Barcelona, Spain; 7 Center for Anti-Infective Agents, Vienna, Austria; 8 Institut für Infektionsprävention und Klinikhygiene, SLK Klinikum, Bad Friedrichshall, Germany

**Keywords:** Ventilation-associated pneumonia, VAP, Bundle, Guideline, Surveillance

## Abstract

**Background:**

To analyse whether the availability of written standards for management of mechanically ventilated patients and/or the existence of a surveillance system for cases of ventilation-associated pneumonia (VAP) are positively associated with compliance with 6 well-established VAP prevention measures.

**Methods:**

Ecological study based on responses to an online-questionnaire completed by 1730 critical care physicians. Replies were received from 77 different countries, of which the majority, i.e. 1351, came from 36 European countries.

**Results:**

On a cross-country level, compliance with VAP prevention measures is higher in countries with a large number of prevention standards and/or VAP surveillance systems in place at ICU level., Likewise, implementation of standards and VAP surveillance systems has a significant impact on self-reported total compliance with VAP prevention measures (both p < 0.001). Moreover, predictions of overall prevention measure compliance show the effect size of the availability of written standards and existence of surveillance system. For instance, a female physician with 10 years of experience in critical care working in a 15-bed ICU in France has a predicted baseline level of VAP prevention measure compliance of 63 per cent. This baseline level increases by 9.5 percentage points (p < 0.001) if a written clinical VAP prevention standard is available in the ICU, and by another 4 percentage points (p < 0.001) if complemented by a VAP surveillance system.

**Conclusions:**

The existence of written standards for management of mechanically ventilated patients in an ICU and the availability of VAP surveillance systems have shown to be positively associated with compliance with VAP prevention measures and should be fostered on a policy level.

## Introduction

Healthcare-associated infections (HAIs) are considered to be a major risk for hospitalised patients and the cause of substantial increases in morbidity, mortality and costs in European Union (EU) member states. Approximately 7 percent of hospitalized patients acquire an HAI while receiving treatment for medical or surgical conditions [1], and it is estimated that each year approximately 37,000 lives are lost to HAI in the EU alone. Healthcare-associated infections incur an estimated Euro 7 billion in excess healthcare costs per annum in the EU, caused mainly by increased length of hospital stay [2]. HAIs are associated with a variety of causes, including but not limited to use of medical devices such as catheters and ventilators, complications following surgical procedures, transmission of pathogens or antibiotic overuse [3,4]. HAIs are often difficult to treat due to antimicrobial resistance (AMR) of the microorganisms causing them [5]. There is a public health interest in preventing HAIs, as laid out in the European Council recommendation of 9^th^ June 2009 in which HAI prevention measures were adopted as part of patient safety programs and quality improvement initiatives [6,7]. National HAI-prevention programs include establishment of surveillance systems, publication of guidelines and measuring structure and process indicators. Furthermore, some European countries have established public reporting of data on HAI from individual hospitals [8,9]*.*

Pneumonia was the HAI most frequently reported in the ECDC pilot point prevalence survey [1], and is most common in the intensive care unit (ICU) [10,11], where a relevant proportion of patients receive mechanical ventilation. Many studies provide evidence for the preventive effectiveness of single interventions, leaving the decision-maker with the complex task of selecting the best one [12-16]. Recently, there has been dramatic success in improving the quality of patient care by focusing on the implementation of an entire group or “bundle” of evidence-based preventive practices [17-19]. These bundle approaches achieve better outcomes than individual implementation of single procedures [20-24], and, from the hospital perspective, have shown to be cost-effective [25,26].

The infection prevention and control measures that have been applied in hospitals to reduce ventilator-associated pneumonia (VAP) vary widely, both within and between different countries [27]. A harmonized approach, based on the application of core strategies developed through an evidence-based approach and comprising specific strategies which relate to local characteristics and context e.g., the affected patient case-mix, should result in a more comparable situation. However, consensus is missing on the most effective infection control interventions or the best combination of interventions to reduce VAP in hospitalized patients. Therefore, we performed a survey to define the level of heterogeneity and analyse related outcomes. In detail, we used the information collected in a questionnaire completed by 1730 ICU physicians across Europe and around the world to analyze whether the availability of written standards for the management of mechanically ventilated patients and/or the existence of a VAP surveillance system on ICU-level are positively associated with compliance with a number of well-established VAP prevention measures.

## Methods

### The dataset

The present work is based on a comprehensive internet-based survey of 1730 ICU physicians. The questionnaire was available online from April 1 to September 1, 2012 and was distributed through various channels by the European Society of Intensive Care Medicine, by national ICU societies in Austria, France, Belgium, the Netherlands, Italy, and Greece as well as through the Revista Electronica de Medicina Intensiva, an electronic newsletter on intensive care medicine in Spanish distributed in Spain, Portugal, and Latin America [28]. Accordingly, the underlying response rate of the survey was unknown. Overall, we received replies from 77 different countries worldwide, of which the majority i.e. 1351 replies came from 36 European countries (including Cyprus, Georgia, Turkey and the Russian Federation). Participation in the survey was anonymous and, as no patient-related information but only process indicators were asked, ethical approval was not necessary [29,30]. See Table 1 for details of the countries with most responses (n > 20). The exact details of the overall survey are already described elsewhere [28,31,32]. The present work applies a cross-country comparison of selected survey results in order to analyse factors influencing compliance with a number of well-established VAP prevention measures. Essentially, the survey generated information about (1) country of abode, (2) some aspects about the hospital in which the physician works, (3) a large number of parameters relating to the ICU in which the physician works and (4) a number of person-specific parameters. With respect to the ICU level, two aspects of the survey are of particular interest for this analysis: Firstly, the physicians were asked to answer the following question (yes/no): “In my ICU, we have written clinical guidelines for the prevention of ventilator-associated pneumonia” (in the following referred to as existence of VAP-standard). Secondly, the physicians answered the question (yes/no) “In my ICU, we count and record VAP on a routine basis” (in the following referred to as existence of VAP surveillance system). Thirdly, the physicians were asked whether, care for intubated patients in their ICU included the following measures: (1) Head of bed elevation; (2) Daily sedation and weaning protocol; (3) Oral care with chlorhexidine; (4) No ventilator circuit change unless indicated; (5) Cuff pressure control at least every 24 hrs; (6) Strict hand hygiene using alcohol, especially before managing the airways (in the following referred to as compliance with prevention measure (1) – (6)). In addition to these 6 specific VAP prevention measures, we determined a variable of overall compliance (compliance-score) by simply adding up self-reported compliance with the 6 specific measures. If for instance all 6 measures are applied in the particular ICU, this variable is equal to 6; if however only two measures are applied the variable is equal to 2.

**Table 1:** Non-representative country averages for countries with >20 responses

**Country**	**Number of responses**	**Number of pseudo-ICUs**	**Mean years of experience in critical care**	**Proportion female sex**	**Number of beds in ICU**	**Availability of VAP prevention standards**	**Existence of VAP surveillance system**	**VAP measure 1**	**VAP measure 2**	**VAP measure 3**	**VAP measure 4**	**VAP measure 5**	**VAP measure 6**	**Sum of VAP measures 1-6**
AR	40	24	15.00	25%	13.20	65%	68%	100%	63%	73%	98%	78%	78%	4.88
AT	130	58	12.75	35%	8.97	40%	45%	96%	44%	47%	93%	98%	82%	4.59
AU	23	17	14.17	9%	19.43	30%	30%	100%	48%	39%	70%	100%	91%	4.48
BE	33	25	17.21	24%	21.15	73%	48%	100%	52%	91%	55%	97%	94%	4.88
BR	21	18	13.86	10%	20.71	81%	76%	100%	71%	86%	95%	62%	67%	4.81
CH	29	16	9.90	24%	12.93	69%	38%	97%	69%	72%	86%	86%	93%	5.03
CO	31	19	9.45	29%	14.84	68%	74%	97%	55%	84%	87%	77%	97%	4.97
DE	67	49	11.81	15%	22.93	67%	52%	97%	54%	60%	93%	99%	84%	4.85
ES	293	107	16.10	38%	15.80	81%	74%	98%	43%	94%	93%	93%	87%	5.09
FR	251	113	11.50	24%	14.88	47%	61%	96%	19%	55%	69%	90%	94%	4.24
GB	115	57	10.74	22%	17.16	79%	50%	98%	81%	90%	50%	87%	87%	4.92
GR	23	15	11.35	61%	11.00	30%	39%	96%	30%	83%	61%	52%	74%	3.96
IN	63	44	9.25	6%	23.94	81%	79%	100%	95%	90%	84%	81%	97%	5.48
IT	187	56	15.47	34%	8.34	60%	48%	91%	35%	76%	50%	70%	70%	3.93
MX	31	19	10.97	10%	10.16	52%	68%	97%	42%	39%	87%	90%	81%	4.35
NL	31	22	9.06	16%	20.87	81%	39%	81%	61%	35%	68%	84%	87%	4.16
PE	23	15	14.52	9%	12.96	43%	57%	96%	48%	4%	91%	78%	48%	3.65
PT	50	27	12.46	42%	12.18	58%	68%	100%	38%	80%	68%	78%	84%	4.48

Availability of VAP prevention standards: country averages of positive responses to the question (yes/no): “In my ICU, we have written clinical guidelines for the prevention of ventilator-associated pneumonia”.

Existence of VAP surveillance system: country averages of positive responses to the question (yes/no): “in my ICU, we count and record VAP on a routine basis”.

VAP prevention measure 1: Head of bed elevation; VAP prevention measure 2: daily sedation and weaning protocol; VAP prevention measure 3: Oral care with chlorhexidine; VAP prevention measure 4: No ventilator circuit change unless indicated; VAP prevention measure 5: Cuff pressure control at lease every 24 hrs; VAP prevention measure 6: Strict hand hygiene using alcohol.

Only countries with >20 responses are included in Table 1.

Country appreviations: ES = SPAIN; FR = FRANCE; IT = ITALY; AT = AUSTRIA; GB = UNITED KINGDOM; DE = GERMANY; IN = INDIA; PT = PORTUGAL; AR = ARGENTINA; BE = BELGIUM; CO = COLOMBIA; MX = MEXICO; NL = NETHERLANDS; CH = SWITZERLAND; AU = AUSTRALIA; GR = GREECE; PE = PERU; BR = BRAZIL.

Although there is no universally accepted gold standard for prevention of VAP, a recent study defined a European care bundle for prevention of VAP and ranked VAP prevention measures by combining criteria such as the strength of the supporting evidence, ease of implementation and expected impact on VAP incidence [33]. The top five clinical interventions of this ranking were included in our questionnaire (VAP prevention measure 2–6). As a control, the most commonly recommended clinical practice of *head of bed elevation* was included in the survey and analyzed as VAP prevention measure 1. Accordingly, self-reported compliance with the target VAP prevention measures were interpreted as compliance with bundle-like VAP prevention measures under routine conditions.

### Statistical analysis

The statistical analysis consisted of three steps. In a first step, country-specific averages of stated compliance with specific VAP prevention measures univariately regressed against the country-average responses to the questions on whether there were written clinical guidelines in place on ICU level for prevention of VAP (and whether there were VAP surveillance systems in place). The results provide a first insight into the relationship between described variables on a cross-country level. Next, within-country averages of stated compliance with the VAP prevention measures were calculated separately for physicians stating whether their ICU had written VAP-standards or not (and whether they had VAP surveillance systems in place or not). Thus, the evidence from these within-country differences in average compliance with the VAP prevention measures was summarized by a p-value from a paired Student’s t-test. In a further step, we endeavoured to utilize the information on inter-ICU differences, i.e. on whether associations exist at ICU-level within each country. Unfortunately, the ICU of each participant is not known. Because our main outcome was defined at ICU level – i.e. compliance with 6 specific measures, we were forced to define pseudo-ICUs by analysing patterns in the ICU characteristics reported by the participants (country of abode, ICU type, number of beds in ICU and number of beds in hospital) and then grouping participants with similar patterns into pseudo-ICUs. This was done in a rather liberal manner to ensure that each existing ICU was covered by a pseudo-ICU, at the same time allowing for pseudo-ICUs to cover several responses. For ICU type and number of beds in hospital only three answers were possible (ICU type: medical, surgical, mixed; number of beds in hospital: < 300, 300–1000, > 1000) while number of beds in an ICU was a continuous variable. The number of ICUs we were able to define this way in the different countries is shown in Table 1. Within each pseudo-ICU we have some variation in the individual responses, which both reflects the flaws in the pseudo-ICUs and the differences in the individual responses within an ICU. We therefore decided to perform an analysis at the individual level, taking however both country and pseudo-ICU levels into account to avoid an overoptimistic assessment of the statistical significance since we had ignored the high correlations between the outcomes within each ICU. Moreover, using pseudo ICUs instead of the true ICUs results in more valid p-values here because possible and to some extent unknown clustering is taken into account. We used linear regression/logistic regression models with the covariates of interest, adjusting for country (as a categorical covariate) as well as number of beds, gender and experience as individual measurements, and took the clustering within pseudo ICUs into account by using robust standard errors (application of the cluster option in Stata 12). Using pseudo ICUs instead of ICUs is a valid approach here because the central assumption of independence of the outcomes between clusters is still valid, even if the clusters are bigger than necessary. As covariates of interest we included presence of written clinical standards on ICU level and presence of VAP surveillance systems.

## Results

### Standard existence and compliance with VAP prevention measures

**Figure 1:**
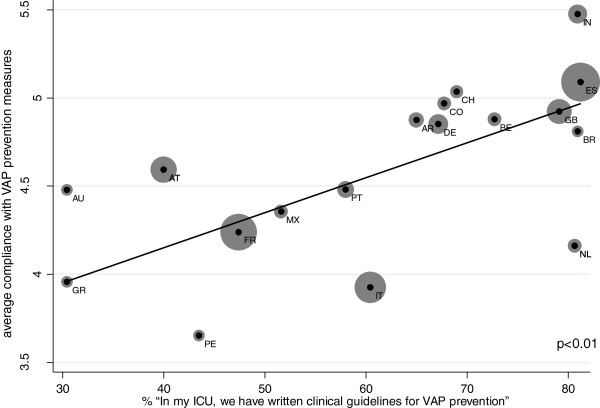
Mean VAP Guideline existence and compliance with VAP preventione measures between countries.

As shown in Figure 1, country averages for countries where there were more than 20 responses of self-reported compliance with the different VAP prevention measures are positively associated with the country-averages of self-reported presence of written clinical standards for prevention of VAP on the ICU level (p < 0.01).

**Figure 2:**
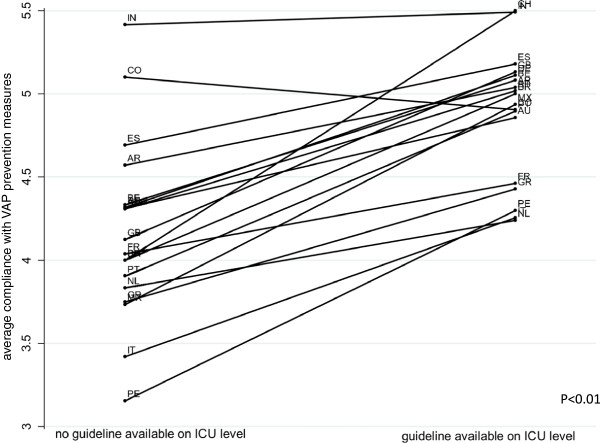
Mean VAP Guideline existence and self-reported compliance with VAP prevention measures within countries.

Furthermore, Figure 2 shows that in most countries where there were more than 20 responses, average self-reported compliance with the different VAP prevention measures is higher when the respondents also confirmed presence of written VAP prevention standards on ICU level (p < 0.01). Correspondingly, we can postulate a connection between compliance with VAP prevention measures and the presence of VAP prevention standards on the between-country (Figure 1) and within-country (Figure 2) level.

As shown in Additional file 1: Figure S1 (available as supplemental material), the results displayed by individual prevention measures provide mixed results. VAP prevention measure 1 - *head of bed elevation* - is commonly recommended clinical practice with high rates of compliance; however, when looking at the between-country or within-country averages there does not seem to be an obvious connection with the presence of VAP prevention standards. Regarding VAP prevention measure 2 and 3, which refer to *daily sedation vacation and weaning protocol* and *oral care with chlorhexidine*, there seems to be a connection between the respective prevention measure and the existence of VAP prevention standards when regarding the between- country and within- country averages. Regarding VAP prevention measure 4 and 5, which refer to *no ventilator circuit change unless indicated* and *cuff pressure control at least every 24 hrs*, the country-averages show no clear connection. VAP prevention measure 6, which represents *strict hand hygiene using alcohol, especially before managing the airways*, shows quite a strong association when looking at the within-country averages, but absolutely no association when looking at the between-country averages.

### Existence of VAP surveillance system and compliance with VAP prevention measures

**Figure 3:**
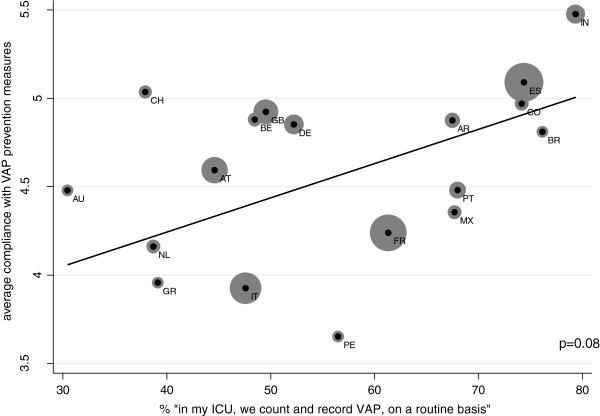
Mean VAP surveillance system existence and self-reported compliance with VAP prevention measures between countries.

As shown in Figure 3, country averages of self-reported compliance with the different VAP prevention measures are positively associated with the country averages of self-reported existence of a VAP surveillance system at the 10 percent level only (p = 0.08).

**Figure 4:**
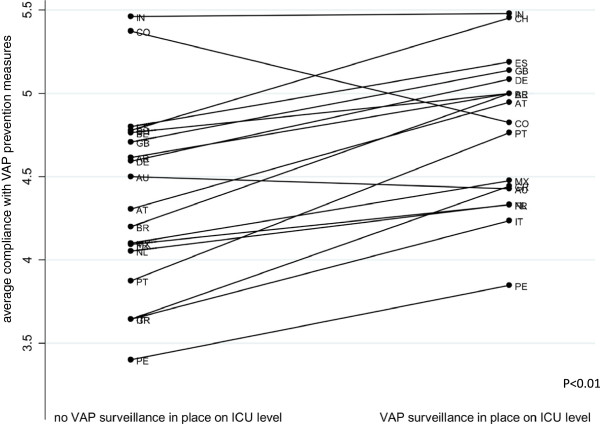
Mean VAP surveillance system existence and self-reported compliance with VAP prevention measures within countries.

On the other hand, the connection between prevention measure compliance and existence of a surveillance system appears to be more obvious on the within-country level (See Figure 4).

### Results of statistical analysis at the individual level

The regression analyses applied take both the interrelation within and between countries into account and show a significant independent association between most of the VAP prevention measures and the existence of written VAP prevention standards on ICU level (see regression (1) to (6) in Table 2). Please note that the non-significance with respect to VAP prevention measure 1 may be a result of the ceiling effect since compliance with the commonly recommended clinical practice of *head of bed elevation* is very high (see Table 1). The existence of a VAP surveillance system, however, has a slightly smaller impact on compliance with the specific measures. Column (7) in Table 2 shows the results of the analysis in which the variable of total compliance (compliance-score) is placed on the left-hand side of the regression analysis. In detail, the analyses show that existence of standard and VAP surveillance systems impact substantially on total compliance with the VAP prevention measures (both p < 0.001). The country-specific implications of regression (7) may be interpreted as follows: According to our model, a female physician with 10 years of experience in critical care working in a 15-bed ICU in France for instance has a predicted baseline level of VAP prevention measure compliance (compliance-score) of 4.309-0.0329 + (10*0.00594) + (15*0.00316)-0.598 = 3.7849, which equals 63%. This baseline level of compliance with VAP prevention measures increases by 0.573 (p < 0.001) if a written clinical VAP standard is available on ICU level, and by 0.244 (p < 0.001) if a VAP surveillance system is in place, which is equivalent to a 15% and 6% increase in the level of compliance with VAP prevention measures, respectively.

**Table 2:** Results of statistical analyses at the ICU level

	**(1)**	**(2)**	**(3)**	**(4)**	**(5)**	**(6)**	**(7)**
	**Logistic regression VAP measure 1**	**Logistic regression VAP measure 2**	**Logistic regression VAP measure 3**	**Logistic regression VAP measure 4**	**Logistic regression VAP measure 5**	**Logistic regression VAP measure 6**	**Linear regression VAP measures 1-6**
Availability of VAP guidelines on ICU level	1.803	2.721^***^	2.137^***^	1.336^*^	1.456^*^	2.847^***^	0.573^***^
Existence of VAP surveillance system on ICU level	1.722	1.320^*^	1.434^**^	1.001	1.403^*^	1.908^***^	0.244^***^
Years of experience in critical care	1.037	1.004	0.992	1.018^*^	0.996	1.032^***^	0.00594^*^
Female sex	0.874	0.919	1.303	0.677^**^	0.987	1.095	-0.0329
Number of beds in ICU	0.987	0.996	1.022^*^	1.013	0.992	1.009	0.00316
ES	1	1	1	1	1	1	0
AR		2.747^**^	0.189^***^	3.179	0.265^**^	0.617	-0.0933
AT	0.681	1.687^*^	0.0842^***^	1.328	3.800^*^	1.421	-0.147
AU		2.333	0.0592^***^	0.173^***^		3.397	-0.222
BE		1.685	0.693	0.0783^***^	2.809	2.948	-0.126
BR		3.506^**^	0.339	1.367	0.119^***^	0.267^**^	-0.293
CH	0.720	3.963^***^	0.204^**^	0.531	0.517	3.690	0.146
CO	0.669	1.924	0.343	0.582	0.254^**^	6.657	-0.00351
DE	0.851	1.982^*^	0.0889^***^	0.885	5.765	1.061	-0.108
FR	0.704	0.426^***^	0.0958^***^	0.190^***^	0.803	4.085^***^	-0.598^***^
GB	1.329	6.824^***^	0.563	0.0735^***^	0.531	1.329	-0.0721
GR	0.694	1.062	0.462	0.168^***^	0.101^***^	0.955	-0.704^**^
IN		30.52^***^	0.490	0.356^*^	0.314^*^	4.880^*^	0.382^***^
IT	0.222*	0.908	0.291^***^	0.0844^***^	0.189^***^	0.491^**^	-0.953^***^
MX	0.650	1.277	0.0506^***^	0.584	0.734	1.027	-0.509^**^
NL	0.108**	2.493	0.0299^***^	0.152^***^	0.435	1.482	-0.818^**^
PE	0.506	1.846	0.00364^***^	0.839	0.313^*^	0.198^**^	-1.168^***^
PT		1.024	0.301^**^	0.193^***^	0.278^**^	1.164	-0.425^**^
Constant							4.309^***^
*N*	1270	1668	1676	1670	1621	1641	1730

VAP prevention measure 1: Head of bed elevation; VAP prevention measure 2: daily sedation and weaning protocol; VAP prevention measure 3: Oral care with chlorhexidine; VAP prevention measure 4: No ventilator circuit change unless indicated; VAP prevention measure 5: Cuff pressure control at lease every 24 hrs; VAP prevention measure 6: Strict hand hygiene using alcohol. In column (1) to (6), odds ratios of the logistic regression models are shown; Column (7) shows non-exponentiated regression coefficients of the linear regression model that was fitted by the least squares approach. In the regression analyses, all 77 countries were included as categorical variables. For lack of space, however, only those for countries with most responses (n > 20) are shown in the present table. The clustering within pseudo ICUs was taken into account by using robust standard errors (application of the cluster option in Stata 12). Country appreviations: ES = SPAIN; FR = FRANCE; IT = ITALY; AT = AUSTRIA; GB = UNITED KINGDOM; DE = GERMANY; IN = INDIA; PT = PORTUGAL; AR = ARGENTINA; BE = BELGIUM; CO = COLOMBIA; MX = MEXICO; NL = NETHERLANDS; CH = SWITZERLAND; AU = AUSTRALIA; GR = GREECE; PE = PERU; BR = BRAZIL.

^*^p < 0.05, ^**^p < 0.01, ^***^p < 0.001.

## Discussion

Our results indicate that the availability of written guidance documents (we do not know whether they were really guidelines or internal guidance documents) to prevent VAP in ICU patients is significantly associated with compliance with the prevention measures. A number of interventional studies exist that analyse adherence with all elements of previously defined ventilator bundles [19,25,26,34,35]. In a comparative approach, Bouadma et al. [23] analysed the preventive impact of increased compliance with backrest elevation, tracheal cuff pressure maintenance, orogastric tube use, gastric overdistension avoidance, good oral hygiene and nonessential tracheal suction elimination in a 20-bed medical ICU in a teaching hospital in France [23]. The authors define a composite score of compliance with the different measures (range, 0–6), and show that after implementation of the bundle in their ICU, the score significantly increased over time, while the VAP prevalence rate decreased [23]. Although the present analysis also uses a score of compliance with prevention measures, the focus however is on the question of why compliance scores differ across ICUs in the absence of specific interventions. Interestingly, our results also point out the positive impact of surveillance systems on compliance with VAP prevention measures. Although the cause-and-effect chain between surveillance and prevention measure compliance is still unclear, the efficacy of surveillance systems in the prevention of hospital acquired infections has been shown previously [36-39]. Moreover, the results also show the heterogeneity among European and non-European countries in the level of compliance with VAP prevention measures. According to the evidence presented here, this heterogeneity may to some degree be explained by the heterogeneity in the availability of standards and the existence of surveillance system. The ICU-specific cause-and-effect relationship between availability of standards, and/or the existence of surveillance system and compliance with VAP prevention measures, however, is still unclear. Hence, we cannot exclude that the availability of standards (and/or surveillance system existence) might be a result rather than a cause of a high level of awareness of VAP, which, in turn, results in a high level of compliance with VAP prevention measures. We may only conclude that on average, ICUs where standards are available and surveillance systems are in place report a significantly higher level of compliance with VAP prevention measures irrespective of national compliance levels.

It should be noted that there are a number of limitations to the work we present that must be taken into account. Firstly, to preserve the respondents’ anonymity, the online questionnaire did not include questions allowing for identification of the ICU [28]. Accordingly, we were faced with the problem that the participant’s identity was unknown. Secondly, the survey did not include randomised sampling, meaning that some categories of ICU physician might have been overrepresented and that the country-averages shown in Table 1 cannot be generalized. Thirdly self-reported compliance might also have been subject to overreporting. Finally, we do not know what type of document the respondents were referring to when confirming the availability of standards on the ICU level. Overall, there was no possibility to validate any of the 1730 responses.

## Conclusions

This study shows wide variability in compliance with VAP-preventive measures across ICUs in Europe. However, two things seem to be of special interest for improvement of compliance: The presence of written standards for management of mechanically ventilated patients and existence of an established VAP surveillance system. These two basic IPC measures should be fostered on a policy level.

## Competing interests

This work was part of the IMPLEMENT project (http://www.eu-implement.info/) which was supported by the European Commission’s Directorate General for Health and Consumers (Grant Agreement N° 20091107). There are no competing interests to declare.

## Authors’ contributions

KK conceived of the study, carried out the statistical analysis and drafted the manuscript. M-LL developed and distributed the questionnaire, collected the data and revised the manuscript critically. UF, JR, MW, ET and UT made substantial contribution in interpreting the results of statistical analyses and revised the manuscript critically. WV participated in the statistical analysis and revised the manuscript critically. MM helped to conceive the study, was involved in drafting the manuscript and revised the manuscript critically. All authors read and approved the final manuscript.

## Pre-publication history

The pre-publication history for this paper can be accessed here:

http://www.biomedcentral.com/1471-2334/14/199/prepub

## Supplementary Material

Additional file 1: Figure S1 VAP Guideline existence and self-reported preventione measure compliance within and between countries. Click here for file
