# Supplementary material for: Impact of availability of guidelines and active surveillance in reducing the incidence of ventilator-associated pneumonia in Europe and worldwide
Source: BMC Infect Dis. 2014 Apr 12;14:199. doi: 10.1186/1471-2334-14-199 (PMC4021349; doi:10.1186/1471-2334-14-199)
Supplement: Additional file 1: Figure S1 — VAP Guideline existence and self-reported preventione measure compliance within and between countries. [file 1471-2334-14-199-S1.docx]

| Figure S1: VAP Guideline existence and self-reported preventione measure compliance within and between countries | | |
| --- | --- | --- |
|  | Guideline existence and self-reported measure compliance within a country | Guideline existence and self-reported measure compliance between countries |
| A |   p=0.025 |   p=0.744 |
| B |   p<0.01 |   p<0.01 |
| C |   p<0.01 |   p=0.025 |
| D |   p=0.029 |   p=0.752 |
| E |   p=0.257 |   p=0.914 |
| F |   p<0.01 |   p=0.261 |
|  | On the ordinate: Within-country averages of the compliance with the respective VAP prevention measures as stated as being included in the routine care of intubated patients; Responses were distinguished into two groups (see abscissa) depending on the answer to the question (yes/no): “In my ICU, we have written clinical guidelines for the prevention of ventilator-associated pneumonia”. Only countries with >20 replies were included in Figure 2 and Student’s T-test. P-value of a paired Student’s T-test is shown in the Figures. | On the ordinate: Country averages of the respective VAP prevention measure as stated as being included in the routine care of intubated patients; On the abscissa: Country averages of positive responses to the question (yes/no): “In my ICU, we have written clinical guidelines for the prevention of ventilator-associated pneumonia”. Only countries with >20 replies were included in Figure 1 and univariate linear regression analysis. P-values of a univariate ordinary least squares regression analysis are shown in the Figures. |
|  | VAP prevention measure 1: Head of bed elevation; VAP prevention measure 2: daily sedation and weaning protocol; VAP prevention measure 3: Oral care with chlorhexidine; VAP prevention measure 4: No ventilator circuit change unless indicated; VAP prevention measure 5: Cuff pressure control at lease every 24hrs; VAP prevention measure 6: Strict hand hygiene using alcohol. | |
